# Dataset of annual metal scrap circularity of titanium industry in China from 2005 to 2020

**DOI:** 10.1038/s41597-023-02351-4

**Published:** 2023-07-06

**Authors:** Wenhao Wang, Fuzhong Wu

**Affiliations:** 1 School of Materials & Metallurgy, Guizhou University, Guiyang, 550025 China; 2 Present Address: School of Materials & Metallurgy, Guizhou University, Guiyang, 550025 China

**Keywords:** Industry, Statistics

## Abstract

Titanium products, regarded as a strategic metal by many national governments, play important and irreplaceable roles in national defence and military applications. China has built a large-scale titanium industrial chain, and its status and development pathways will greatly affect the global market. Several researchers contributed a set of reliable statistical data to bridge the knowledge gap in evaluating the industrial layout and the entire structure of China’s titanium industry with little literature information regarding the management of metal scrap in the manufacturers of titanium products. To bridge this data gap, we present a dataset of annual metal scrap circularity to uncover China’s evolution of the titanium industry today, which contains off-grade titanium sponge, low-grade titanium scrap, and recycled high-grade titanium swarf with the relevant circularity of the titanium industry in China at the national level from 2005 to 2020.

## Background & Summary

Titanium alloys primarily stand out due to their high specific strength and excellent corrosion resistance, which explains their preferential and critical applications in the aerospace industry, national defence, and military applications as the key structural materials for the long-term service^1,2^. Therefore, there are stern technological barriers to the manufacture of titanium and titanium-based alloys, while they are regarded as strategic resources by many national governments^1^.

**Fig. 1:**
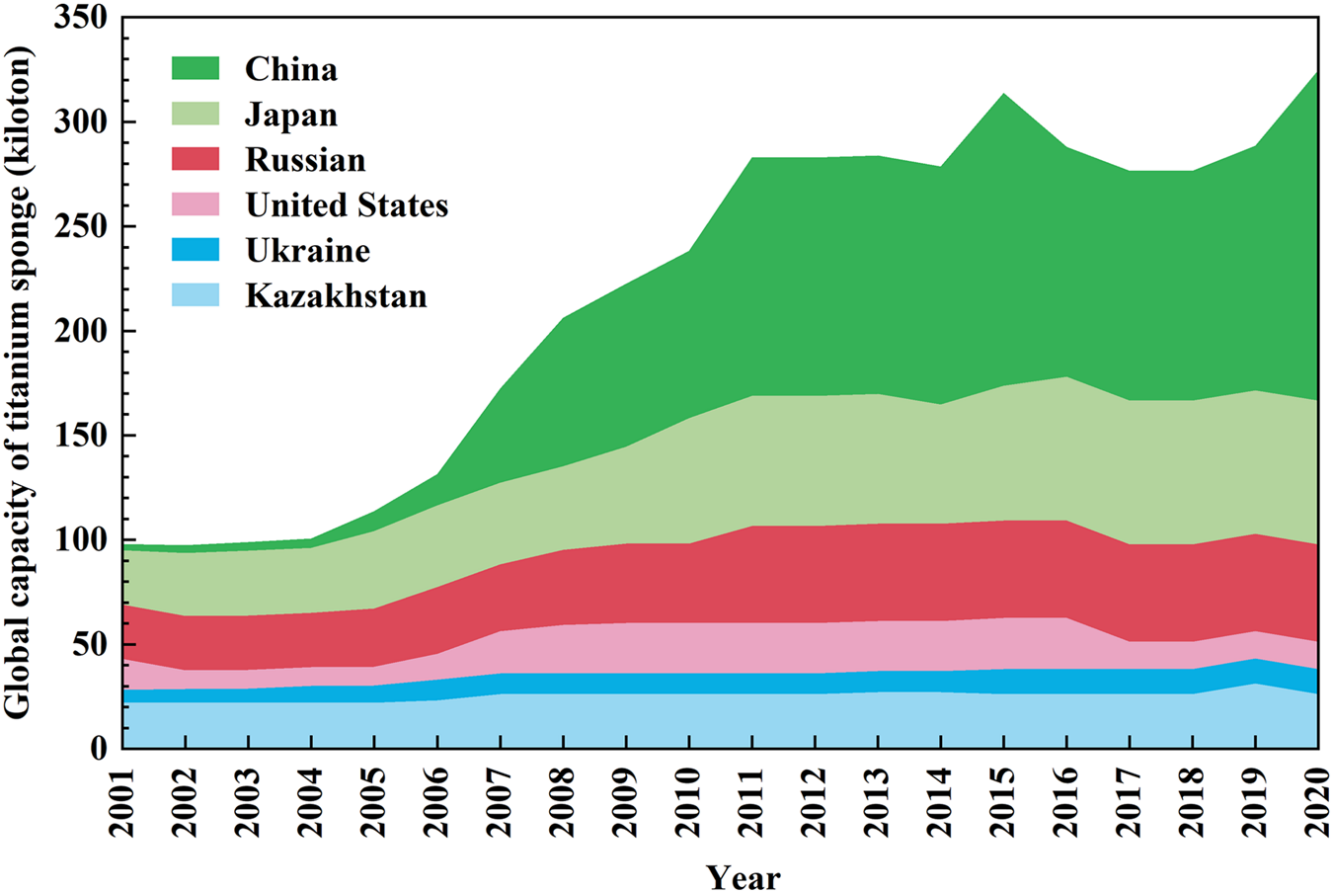
Global production capacity of titanium sponge from 2001 to 2020.

China plays a tiny role in the global titanium market, and it only shares (0.8~4.5%) of the worldly titanium sponge production capacity from 1995 to 2004^3,4^. And China’s titanium industry has experienced significant development to meet its outstanding economic performance and become a decisive titanium producer and consumer globally since 2005. Its share in the global production capacity of titanium sponge, the primary titanium metal, increased from 8.4% in 2005^5^ to 48.7% in 2020^6^, followed by Japan (21.2% in 2020), Russian (14.3% in 2020), Kazakhstan (only 8.0% in 2020), US (only 4.0% in 2020), and Ukraine (only 3.7% in 2020)^6^, and they cover the current global titanium sponge requirement, which detailed in Fig. 1. And China also produced about 123.0 thousand tons of titanium sponge in 2020, which is responsible for more than 55.0% of the overall titanium sponge production^7^. China has been a dominant role in the global titanium supply chain. China also experiences rapid growth in titanium alloy products, while its average annual growth rate exceeds 16.5% from 2001. And China has also been the main consumer of the global titanium consumption chain. Meanwhile, the annual growth rate of internal demand for titanium alloy products averaged at about 20.0% from 2001 and shared 46.0% of the global demand for those products in 2020. This rapidly rising caused by the aerospace industry and the national defence is likely to continue to grow in the next few decades, which put new demands on the supply security of titanium products. Thus, the status and development pathways of China’s titanium industry will have a significant impact on the market of the entire world^7,8^.

Although China has built a large-scale titanium industrial chain, it is still weak in the high-end titanium-based alloys and needs urgent upgrade^8,9^. And only the individual regional governments have put forward several development plans and policies to regulate their titanium industries, few policies have yet been carried out at the national level to explore the development strategy of China’s titanium industry and related industries in strengthening resource management, low carbon and long-term development of high technology. To bridge the knowledge gap in evaluating the industrial layout and the entire structure of China’s titanium industry, several researchers contributed a set of reliable statistical data^7,10^ that are the basis for setting the national development strategy of China’s titanium industry. However, they performed little literature information regarding the management of metal scrap in the manufacturers of titanium products, which would lead to increased uncertainty in the material flow analysis for titanium sponge. There are also few attempts to quantify waste management and metal scrap circularity within the titanium industry chain in China^7,9–11^. The management of titanium metal scrap would be particularly vital to expand the virgin titanium metal resources and be beneficial to optimize China’s resource efficiency^11^, which is also valuable to resource economists and regional strategic policymakers.

Given the importance of quantifying metal scrap and its circularity, we present a dataset of annual metal scrap circularity to uncover China’s evolution of the titanium industry today, which contains unrecycled low-grade titanium scrap and recycled high-grade titanium swarf with the relevant circularity at the national level in China from 2005 to 2020.

## Methods

### Boundary definition

The spatial boundary of this dataset covers the whole territory of mainland China, except the Hongkong, Macau, and Taiwan regions^12,13^. For periods earlier than the year 2005, the statistical data on the dataset of titanium products are incomplete and China plays a tiny role in the global titanium products market. Thus, the temporal boundary refers to the period of 2005 to 2020 to ensure the integrity and authority of the data collecting in this descriptor.

### Titanium products and titanium scrap

Titanium products fall into four categories according to their life cycle: titanium sponge, titanium ingots, titanium mills, and titanium goods. Titanium tetrachloride reacts with the liquid magnesium and then is refined by vacuum distillation for the removal of non-titanium contaminants, and forming a sponge structure^14,15^. The final product is named as titanium sponge. A considerable amount of the off-grade titanium sponge, which constitutes 10.0% to 20.0% of the annual production, is generated and currently used as an alloying addition to titanium-stabilized specialty steels, either directly recycled into ferrotitanium^16,17^.

Then, titanium sponge and small-piece swarf metal are firstly pre-densified in a hydraulic press and then assembled to an electrode that is at least double re-melted under low-pressure argon in an electron beam melting furnace or a vacuum consumable arc remelting furnace^18^. The slabs, bars, and flat-rolled materials formed by forging are the semi-finished products for the rolled slabs and plates in rolling facilities^19^. In this study, the intermediate products, called titanium mills, include more than six categories of slabs & plates, bars, tubes, wires, forged pieces, castings, and the other mill products.

Titanium mills are further processed to manufacture end-use products. The application scenarios for the terminal usage of titanium goods are classified as the chemical industry, aerospace industry, marine industry, metallurgical industry, power industry, medical instruments, salt manufacturing, oceanographic industry, sports & leisure, and others.

The machining processes of titanium ingots and mills generate a large amount of prompt titanium swarf, which accounts for about 25.0% to 40.0% of titanium ingots production. Furthermore, the high-grade titanium swarf with low O and Fe impurities is remelted to titanium ingots or even slabs. And low-grade swarf, about 10.0% to 25.0% of that production, would be also used as an alloying element in the steel industry^20^. Due to the fact that the majority of the titanium embodies is still under service, but the backward recycling technology, the volume of end-of-life scrap is quite low at less than 1.0% and its recycling is not important on an industrial scale at this stage in China^19^. The various lifetime of end-use products has little impact in determining the metal scrap circularity at the present stage. Thus, the major resource of recycled titanium scrap is generated in the manufacturing process of titanium ingots and mills, rather than the post-consumer in-use titanium products.

**Fig. 2:**
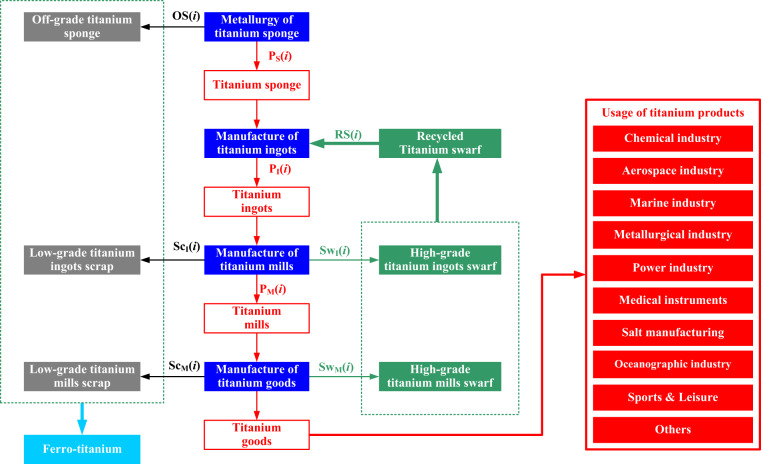
Life cycle of titanium products and titanium scrap management & recycling.

The details of titanium products and relevant titanium scrap, off-grade titanium sponge, low-grade titanium scrap, and high-grade titanium swarf, are displayed in Table 1 and Fig. 2.

**Table 1:** List of titanium products and relevant titanium scrap.

Products name	Products categories	Titanium scrap
Titanium sponge	Titanium sponge	Off-grade titanium sponge
Titanium ingots	Titanium ingots	
Titanium mills	Slabs & Plates; Bars; Tubes; Wires; Forged pieces; Castings; Other mills	Low-grade titanium ingots scrap; High-grade titanium ingots swarf
Titanium goods	User defined titanium goods	Low-grade titanium mills scrap; High-grade titanium mills swarf

### Assessment of titanium scrap and relevant circularity

The total of recycled titanium swarf and assessment of titanium scrap circularity is estimated in Table 2.

**Table 2:** Assessment of titanium scrap and relevant circularity.

Life cycle	Inputs/Outputs	Equation
Metallurgy of Titanium sponge	P_S_(*i*): Domestic production of titanium sponge OS(*i*): Off-grade titanium sponge	
Manufacture of Titanium ingots	P_I_(*i*): Domestic production of titanium ingots RS(*i*): Recycled high-grade titanium swarf	RS(i) = Sw_I_(i) + Sw_M_(i)
Manufacture of Titanium mills	P_M_(*i*): Domestic production of titanium mills Sc_I_(*i*): Low-grade titanium ingots scrap Sw_I_(*i*): High-grade titanium ingots swarf	
Manufacture of Titanium goods	Sc_M_(*i*): Low-grade titanium mills scrap Sw_M_(*i*): High-grade titanium mills swarf	
Titanium scrap & circularity	OSR(*i*): Ratio of off-grade titanium sponge to domestic production ScR_I_(*i*): Ratio of low-grade titanium ingots scrap to domestic production of titanium ingots SwR_I_(*i*): Ratio of high-grade titanium ingots swarf to domestic production of titanium ingots ScR_M_(*i*): Ratio of low-grade titanium mills scrap to domestic production of titanium ingots SwR_M_(*i*): Ratio of high-grade titanium mills swarf to domestic production of titanium mills RR(*i*): Recycling rate of titanium scrap	OSR(*i*) = OS(*i*)/P_S_(*i*) ScR_I_(*i*) = Sc_I_(*i*)/P_I_(*i*) SwR_I_(*i*) = Sw_I_(*i*)/P_I_(*i*) ScR_M_(*i*) = Sc_M_(*i*)/P_I_(*i*) SwR_M_(*i*) = Sw_M_(i)/P_I_(*i*) RR(i) = RS(i)/[RS(i) + OS(i) + Sc_I_(i) + Sc_M_(i)]

*NOTE:* Full forms of all abbreviations are next to the abbreviations in this table.

### Data management

**Fig. 3:**
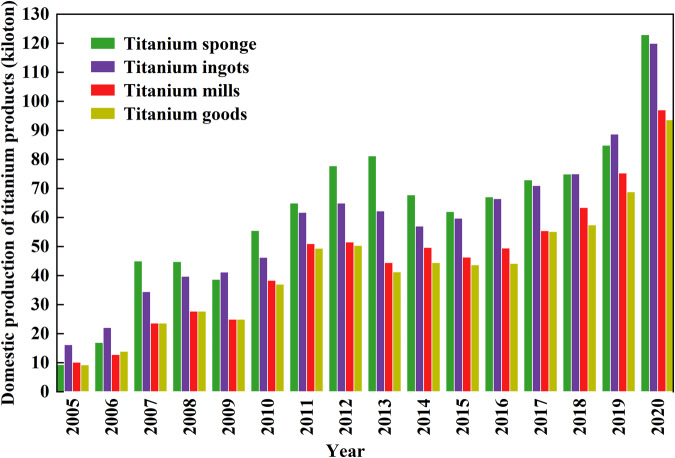
Yearly domestic production of titanium sponge, titanium ingots, titanium mills, and titanium goods from 2005 to 2020.

**Fig. 4:**
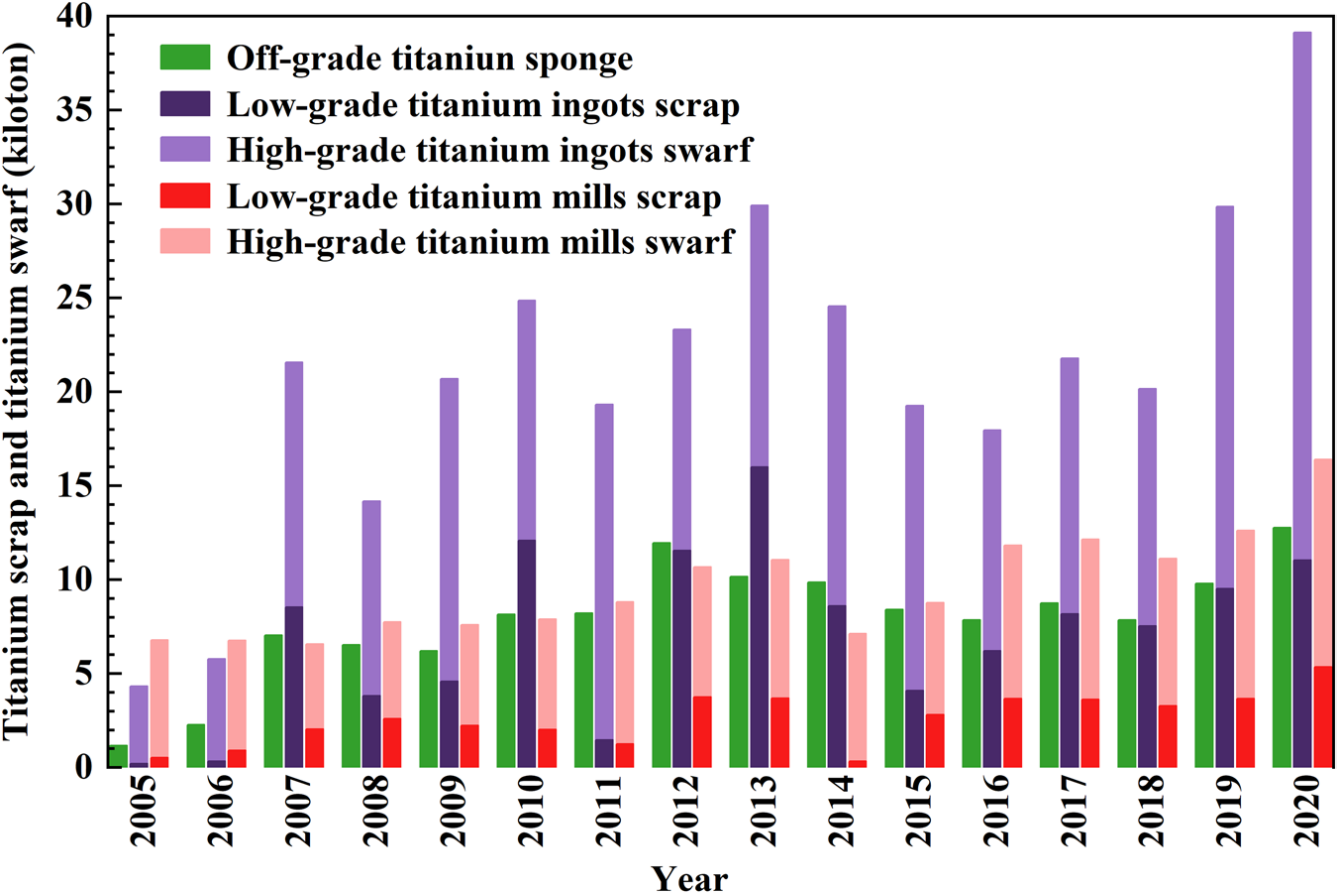
Yearly recycled prompt swarf and unrecycled scrap in China’s titanium products industrial chain from 2005 to 2020.

The yearly domestic production data from 2005 to 2020 are mainly consulted from the published annual literature, the China Non-ferrous Metals Industry Association Titanium Zirconium & Hafnium Branch (CNIA-TI), or the annual publications of the USGS (Fig. 3). For the difference, the general principle is to give a priority to adopting official statistics from Chinese literature and the China Non-ferrous Metals Industry Association. Titanium scrap in different stages in practical production are obtained through our field investigations and consultation with relevant experts (Fig. 4). Those data are regarded as the primary data. The relative uncertainties of primary data are very low, considered to be equal to or less than 2.0%^7^, while those data were collected from official statistical reports and published literature.

The secondary data, recycled high-grade titanium swarf, and the various coefficients are generally calculated from the primary statistics data or the previous reports and estimated by industrial experts. And the resulting relative uncertainties of the secondary data are calculated by error propagation methods, an alternative faster and more reliable than Monte Carlo method within same indicators^7,21^. The details of the data resources and their relevant uncertainties are listed in Table 3.

**Table 3:** Data resources and their uncertainties assessment.

Data		Resources	Relative uncertainty
Primary data	Domestic production: P_S_(i), P_I_(i), P_M_(i) Scrap: OS(i), Sc_I_(i), Sc_M_(i) Swarf: Sw_I_(i), Sw_M_(i)	Chinese literature^23^, CNIA-TI, or USGS^24^ Field investigations	2.0% 2.0% 2.0%
Secondary data	Recycled swarf: RS(i) Ratios: OSR(i) ScR_I_(i), SwR_I_(i), ScR_M_(i), SwR_M_(i) RR(i)	Direct calculation Direct calculation Direct calculation Direct calculation	2.8% 2.8% 2.8% 4.3%

*NOTE:* Full forms of all abbreviations are next to the abbreviations in the Table 2.

## Data Records

The database contains annual primary data and secondary data for titanium products in China from 2005 to 2020. The primary data are recorded as operating capacity, annual production, and titanium scrap of titanium sponge, titanium ingots, titanium mills, and titanium goods. The secondary data are recorded as the recycled high-grade titanium swarf, and the ratios of off-grade titanium sponge, low-grade titanium scrap, and recycled high-grade titanium swarf for each titanium product.

The entire database has been uploaded and publicly available at the *Figshare* repository and is named “Dataset of annual metal scrap circularity of titanium industry in China from 2005 to 2020”^22^, which consisted of five excel files (Table 4).

**Table 4:** Detail of database files.

Number	Name	Details
1	titanium sponge	Operating capacity and annual production of titanium sponge in China during 2005–2020
2	titanium ingots	Operating capacity and annual production of titanium ingots in China during 2005–2020
3	titanium mills	Annual production of titanium mills in China during 2005–2020
4	titanium goods	Internal sales (ten industries) of titanium goods in China during 2005–2020
5	titanium scrap & circularity	Annual production and ratios of the off-grade titanium sponge, low-grade titanium scrap, and recycled high-grade titanium swarf during 2005–2020

## Technical Validation

### Comparison of metal scrap circularity with Japan

**Fig. 5:**
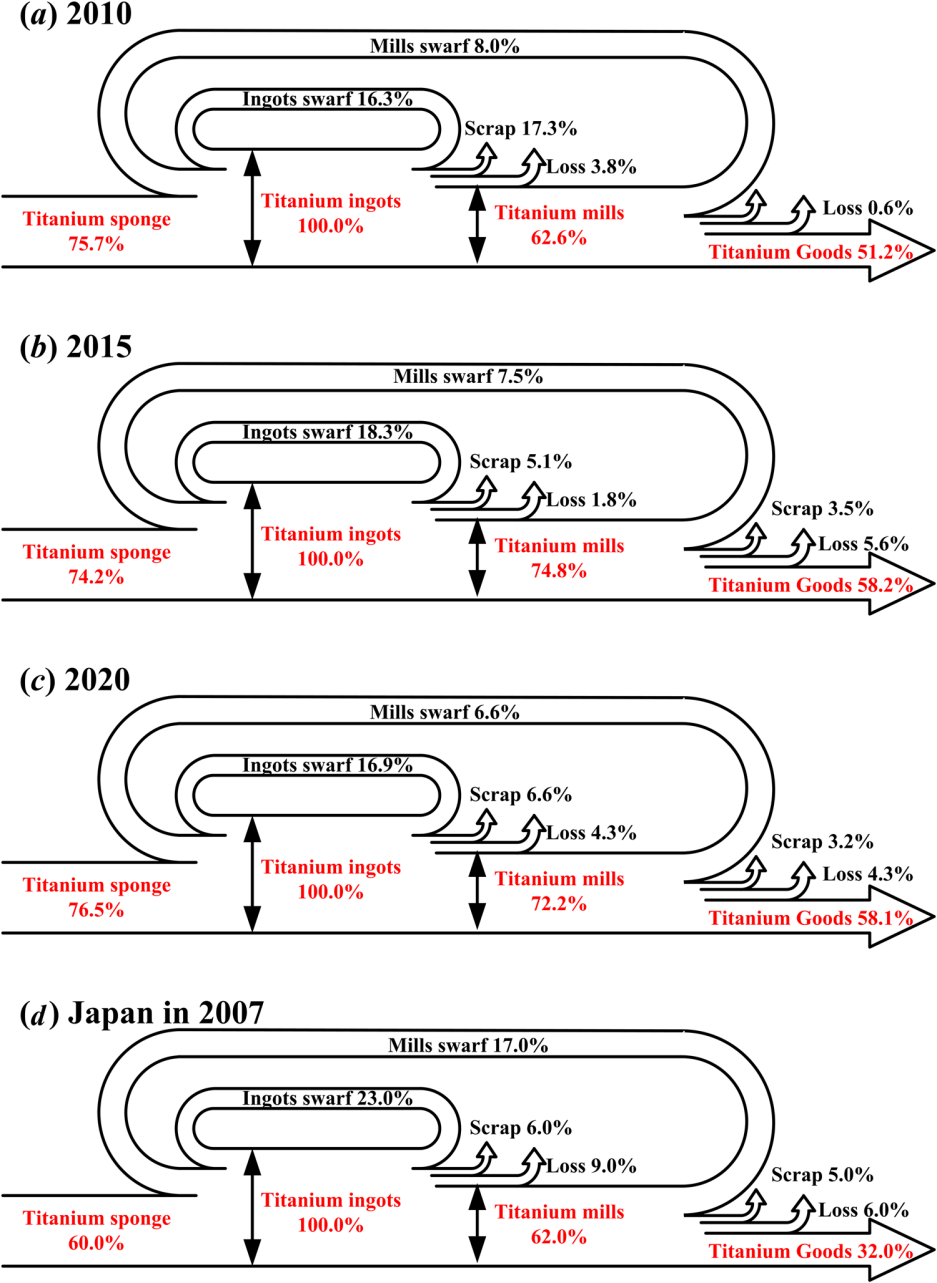
Validation of titanium scrap and swarf for titanium ingots and mills in (**a**) 2010, (**b**) 2015, (**c**) 2020, and that of Japan in 2007.

**Fig. 6:**
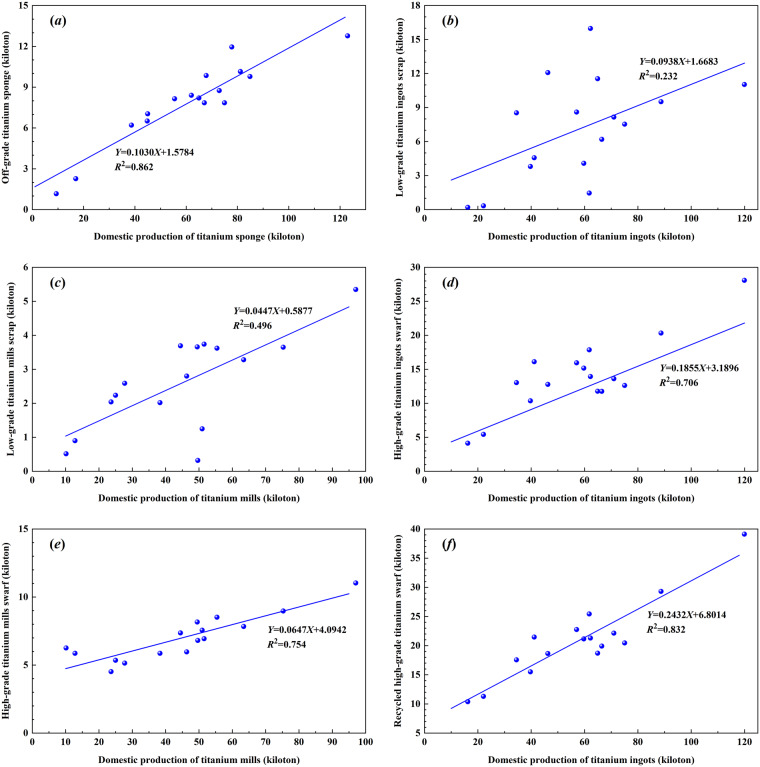
Correlations between titanium scrap and domestic production for (**a**) off-grade titanium sponge, (**b**) low-grade titanium ingots scrap, (**c**) low-grade titanium mills scrap, (**d**) high-grade titanium ingots swarf, (**e**) high-grade titanium mills swarf, and (**f**) recycled high-grade prompt titanium swarf.

Since there is no directly comparable dataset for this validation process, the validation of titanium scrap and swarf for ingots and mills are firstly shown in Fig. 5. The share of recycled prompt swarf keeps on 24.3%, 25.8%, and 23.5% in the raw material supply of titanium ingots manufacturing in years of 2010, 2015 and 2020, respectively. Although the recycled titanium prompt swarf reduces the dependence on high-grade titanium sponge for China to some extent, it is still weak compared with Japan^19^ which keeps on 40% in 2007. Secondly, the off-grade titanium sponge, low-grade titanium scrap, and high-grade titanium swarf demonstrated significant positive correlations with domestic production of relevant titanium products (Fig. 6), which indicated that major uncertainties would not been introduced in this dataset of annual metal scrap and relevant circularity of titanium industry in China from 2005 to 2020.

### Limitations of China’s titanium flow dataset

Great efforts were made to guarantee the reliability of China’s titanium flow dataset, however, the lack of available statistical results for the usage of low-grade titanium scrap for titanium sponge, titanium ingots, and titanium mill products caused that the usages of them were not displayed detailly in this data descriptor. These shortcomings should be considered by users.

## Data Availability

There was no code used in the generation of the data in this work, an only Microsoft Excel is employed to process all the data.
